# Cellular models of development of ovarian high‐grade serous carcinoma: A review of cell of origin and mechanisms of carcinogenesis

**DOI:** 10.1111/cpr.13029

**Published:** 2021-03-25

**Authors:** Jie Mei, Huixiang Tian, Hsuan‐Shun Huang, Che‐Fang Hsu, Yuligh Liou, Nayiyuan Wu, Wei Zhang, Tang‐Yuan Chu

**Affiliations:** ^1^ Department of Clinical Pharmacology Xiangya Hospital Central South University Changsha China; ^2^ Institute of Clinical Pharmacology Hunan Key Laboratory of Pharmacogenetics Central South University Changsha China; ^3^ Engineering Research Center of Applied Technology of Pharmacogenomics Ministry of Education Changsha China; ^4^ National Clinical Research Center for Geriatric Disorders Changsha China; ^5^ Department of Pharmacy Xiangya Hospital Central South University Changsha China; ^6^ Center for Prevention and Therapy of Gynecological Cancers Department of Research Buddhist Tzu Chi General Hospital Hualien Taiwan, ROC; ^7^ Hunan Cancer Hospital The Affiliated Cancer Hospital of Xiangya School of Medicine Central South University Hunan China; ^8^ Department of Obstetrics & Gynecology Buddhist Tzu Chi General Hospital Hualien Taiwan, ROC; ^9^ Department of Life Sciences Tzu Chi University Hualien Taiwan, ROC

## Abstract

High‐grade serous carcinoma (HGSC) is the most common and malignant histological type of epithelial ovarian cancer, the origin of which remains controversial. Currently, the secretory epithelial cells of the fallopian tube are regarded as the main origin and the ovarian surface epithelial cells as a minor origin. In tubal epithelium, these cells acquire TP53 mutations and expand to a morphologically normal ‘p53 signature’ lesion, transform to serous tubal intraepithelial carcinoma and metastasize to the ovaries and peritoneum where they develop into HGSC. This shifting paradigm of the main cell of origin has revolutionarily changed the focus of HGSC research. Various cell lines have been derived from the two cellular origins by acquiring immortalization via overexpression of hTERT plus disruption of TP53 and the CDK4/RB pathway. Malignant transformation was achieved by adding canonical driver mutations (such as gain of CCNE1) revealed by The Cancer Genome Atlas or by noncanonical gain of YAP and miR181a. Alternatively, because of the extreme chromosomal instability, spontaneous transformation can be achieved by long passage of murine immortalized cells, whereas in humans, it requires ovulatory follicular fluid, containing regenerating growth factors to facilitate spontaneous transformation. These artificially and spontaneously transformed cell systems in both humans and mice have been widely used to discover carcinogens, oncogenic pathways and malignant behaviours in the development of HGSC. Here, we review the origin, aetiology and carcinogenic mechanism of HGSC and comprehensively summarize the cell models used to study this fatal cancer having multiple cells of origin and overt genomic instability.

## Graphical abstract

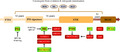

## 1 INTRODUCTION

### 1.1 High‐grade serous carcinoma of the ovary, peritoneum and fallopian tube

Epithelial ovarian cancer (EOC) is one of the most common malignant tumours in women, with approximately 21 750 new cases and 13 940 deaths estimated in the United States in 2020.^1^ The 5 year survival rates have been <45% for many years,^2^ which indicate that either the prevention or the treatment has not improved significantly over the past decades.

Among the different histological types of EOC, high‐grade serous carcinoma (HGSC) is both the most prevalent and most fatal type, accounting for 30%‐60% of cases and 70%‐80% of the mortalities.^3^ The prevalence and high mortality are mainly due to the difficulty of early diagnosis and propensity for recurrence because of resistance to chemotherapeutic agents.^4^, ^5^, ^6^ Consequently, EOC is the 7th most common cause of cancer‐related deaths among women in the world.^1^ Obvious impediments to progress include unclear aetiology, ambiguous tissue of origin and unknown mechanism of malignant transformation.^7^, ^8^, ^9^ Besides the ovary, HGSCs are occasionally found in the fallopian tube and peritoneum as the main lesion.^10^ These extraovarian HGSCs show identical characteristics to the ovarian counterpart, including genetic, molecular and histological features and clinical behaviours,^11^ indicating they are the same disease of different localization.

### 1.2 Ovarian and tubal origin of HGSC, a debated and shifting paradigm

Ovarian surface epithelium (OSE) or cortical inclusion cysts have long been considered to be the orthodox origin of ovarian HGSC.^12^ This dogma was challenged by findings showing that precursor lesions of HGSC termed ‘p53 signature’ and serous tubal intraepithelial carcinoma (STIC) were exclusively found in the epithelium of the fallopian tube, especially at the fimbria part, but not on the ovary.^13^, ^14^, ^15^, ^16^ This shifting paradigm was further reinforced by intensive histopathological analyses, clonality assays of the driver mutations and the whole genome^17^, ^18^, ^19^ of surgical specimens as well as by genetic manipulation in cellular and transgenic mouse models recapitulating the transformation from fallopian tube epithelial cells (FTECs).^20^, ^21^, ^22^ The data from these various investigations point to the secretory cells in fimbrial epithelium as the main cell of origin of HGSC.^23^, ^24^, ^25^ However, this conclusion does not suggest that all HGSCs have a fallopian tube origin. Nostalgic studies involving cellular transformation and a genetic‐engineered mouse model (GEM) keep providing pieces of evidence that HGSC can also arise from OSE.^26^, ^27^, ^28^ The current consensus is that HGSC can arise from both OSE and the fallopian tube, with the fallopian tube epithelium (FTE) as the major origin.^27^, ^29^

### 1.3 Mechanism of transformation: Ovulation‐driven mutagenesis and clonal expansion with loss of progesterone protection

More and more epidemiological studies have supported the theory of incessant ovulation as the cause of HGSC.^30^, ^31^ The hypothesis that ovulation is associated with ovarian cancer was first raised in 1971 by MF Fathalla.^32^ In numerous large‐scale epidemiological studies, the number of ovulations was associated with an increased risk of ovarian cancer in a dose‐dependent manner.^33^, ^34^, ^35^, ^36^ Factors that reduce the number of ovulation cycles, such as pregnancy, lactation and use of oral contraceptives, showed a protective effect on HGSC.^32^, ^37^

The underlying mechanisms of carcinogenesis caused by incessant ovulation were explored under different assumptions. The endocrine mechanism stating a carcinogenic role of gonadotropins and oestradiol, which peak during ovulation and exert a transformation effect,^37^, ^38^, ^39^ was refuted by a study showing that FTECs did not proliferate or display increased DNA damage in response to either oestrogen or follicle‐stimulating hormone/luteinizing hormone.^31^ Alternatively, the tear‐and‐repair hypothesis proposed by Fathalla^32^ states a repetitive proliferation of the OSE after ovulation‐induced wounding,^40^ which increases DNA damage and may enhance the transformation.^41^ It was later noted that without a tearing, fallopian tube fimbria, bathed by follicular fluid (FF) during oocyte catch up, is also vulnerable to ovulation injury, such as inflammation and DNA damage.^42^, ^43^, ^44^, ^45^ Eventually, the focus turned to the contents in the FF that bath both the ovulatory wound and FTE after ovulation. FF contains high concentrations of growth factors, proteinases involving coagulation cascades and others, extracellular proteins, hormones, immune agents and reactive oxygen species (ROS).^46^, ^47^, ^48^, ^49^ Among them, ROS causes tissue injury and DNA double‐strand breaks on the epithelium of fallopian tube fimbria and was regarded as a mutagen in FF,^48^, ^50^, ^51^ and some growth factors such as IGF2 cause clonal expansion and transformation of immortalized FTECs.^48^, ^51^, ^52^

More than the DNA damage, FF also exerts stem cell activation and clonal expansion activity on FTECs. FF contains abundant insulin‐like growth factor (IGF)‐axis proteins, including IGF‐binding protein 2/6, IGF1/2 and the IGF‐binding proteolytic enzyme, pregnancy‐associated plasmatic protein A (PAPP‐A). The expression levels of these proteins increased with the growth of the ovarian follicle.^53^ After ovulation, the IGFBP‐cleavage enzyme PAPP‐A is activated upon tethering to the membrane of FTECs. IGF2 is then released and binds to the adjacent membrane receptor IGF‐1R and activates the AKT/OCT2/ NANOG and AKT/mTOR pathways. These signals of stemness activation and clonal expansion, on the one hand, repair the injury caused by ovulation, and on the other, lead to expansion of cancer initiation cells and malignant transformation.^52^ Thus, a regeneration mechanism is reserved in the ovarian follicle, which is timely and locally activated after ovulation. However, the cost is carcinogenesis of the exposed epithelium of the fimbria and the ovary, a justifiable price following the priorities of reproduction and evolution.

In addition to ovulation, the backflow of menstrual blood or retrograde menstruation may also promote development of ovarian cancer. The risk of ovarian cancer decreased after the retrograde route was blocked by tubal ligation.^54^, ^55^, ^56^ Iron metabolites in menstrual blood exposed to the FTE and ovary may exert the Fenton reaction upon iron oxides by interacting with H_2_O_2_ released from ovulation. It was reported that iron‐transporter protein transferrin, via its receptor TfR1, facilitated formation of DNA double‐strand breaks in FTE.^57^ Moreover, haemoglobin from retrograded menstrual blood readily quenched the excessive ROS released from ovulation and rescued the exposed FTE from the ROS‐induced apoptosis. The surviving FTE under this tolerable ROS stress still accumulates DNA double‐strand breaks and proceeds to transformation.^48^ Therefore, ovulation and retrograde menstruation produce a repeated tolerable Fenton reaction on FTE and OSE, which may promote development of ovarian cancer.^58^, ^59^

Given that both ovulation and retrograde menstruation are almost regular events^60^ but the prevalence of STIC (<1%)^61^ and the lifetime risk of ovarian cancer (about 1 in 78 [https://www.cancer.org/cancer/ovarian‐cancer/about/key‐statistics.html]) are low, early and systemic protection against tubal carcinogenesis is expected. Plethoral pieces of evidence from epidemiological studies have suggested progesterone is the protector. Raised progesterone either from term pregnancy or from use of combine‐ or progestin‐only‐oral contraceptives^62^, ^63^ is associated with a rapid decline of ovarian cancer risk, with an extent far superior to what could be expected when considering ovulation inhibition alone.^64^ A cleansing effect of progesterone specifically on the p53‐defective tubal epithelial cells with sparing of p53‐intact cells was confirmed in Trp53^−/−^ mice and in p53‐mutated or p53‐deficient human FTECs. Progesterone receptor (PR) mediated this cleansing effect by inducing necroptosis via the TNF‐α/RIPK1/RIPK3/MLKL pathway.^65^ Interestingly, PR is downregulated in the most majority of EOCs.^66^ Two polymorphisms at the PGR gene contribute to ovarian cancer susceptibility.^67^ Thus, either intrinsic or extrinsic progesterone protects the development of HGSC at early stage, and loss of PR may be a necessity for the carcinogenesis induced by ovulation and retrograde menstruation.

### 1.4 Molecular pathways involved in the development of HGSC and its precursors in the fallopian tube

Almost all HGSCs and their tubal precursor STICs harbour TP53 mutations,^25^, ^68^, ^69^, ^70^ which are considered to be the first step in the transformation.^71^ The initial TP53 mutation lesion, p53 signature, is a cluster of histologically normal tubal secretory cells with accumulation of mutant p53 in the nucleus, which was estimated to occur about 10 years after the first ovulation.^64^ DNA double‐strand breaks frequently occur in p53 signatures, indicating that it may be induced by ovulation‐ and retrograde menstruation‐related ROS. A more severely transformed tubal epithelial lesion is called STIC, which retains the expression of oviduct secretory cell marker PAX8, TP53 mutation and DNA damage,^72^ and acquires high proliferative activity, cell atypia and loss of cellular polarity. After acquiring metastatic properties, STICs spread to peritoneal organs, including the ovary and peritoneum and becomes clinically evident HGSC.^73^ Interestingly, while STIC has a propensity for intraperitoneal metastasis, it rarely invades deeply into the lamina propria to grow overt fallopian tube cancer. Thus, STIC is found either as an in situ carcinoma or microinvasive carcinoma. Clinically, HGSC mostly presents in the ovary as the primary site rather than in the fallopian tube. We reason that the stroma of fallopian tube must have evolved a mechanism to resist the implantation/invasion of embryo to prevent the fatal ectopic pregnancy. The same mechanism also impedes the invasive growth of STIC.

The molecular relationship among the tubal precursor lesions and HGSC has been clarified by targeted sequencing of the TP53 gene. Mutations were found in 57% of p53 signatures and almost all STICs and HGSCs.^27^, ^74^ Identical TP53 and other mutations were shown in all STIC/ovarian cancer pairs. A recent comprehensive genomic analysis by next‐generation sequencing (NGS) further provided striking evidence that the p53 signature or STIC had an ancestral clonal relationship with HGSC. They shared common driver mutations affecting *TP53, PTEN, BRCA1* or *BRCA2*. Thus, p53 signatures and STICs are precursors of ovarian HGSC. An estimation of the time sequence of their development based on results from epidemiological, molecular and NGS studies suggests 10 years from the normal tubal epithelium to P53 signature, another 15 years from p53 signature to STIC and a final 6+ years from STIC to ovarian HGSC.^64^ Meanwhile, two evolutionary analyses based on the molecular clock of driver mutations in synchronous STIC and HGSC lesions have consistently revealed the sojourn time between STIC and HGSC is 6‐7 years.^17^, ^19^

**FIGURE 1:**
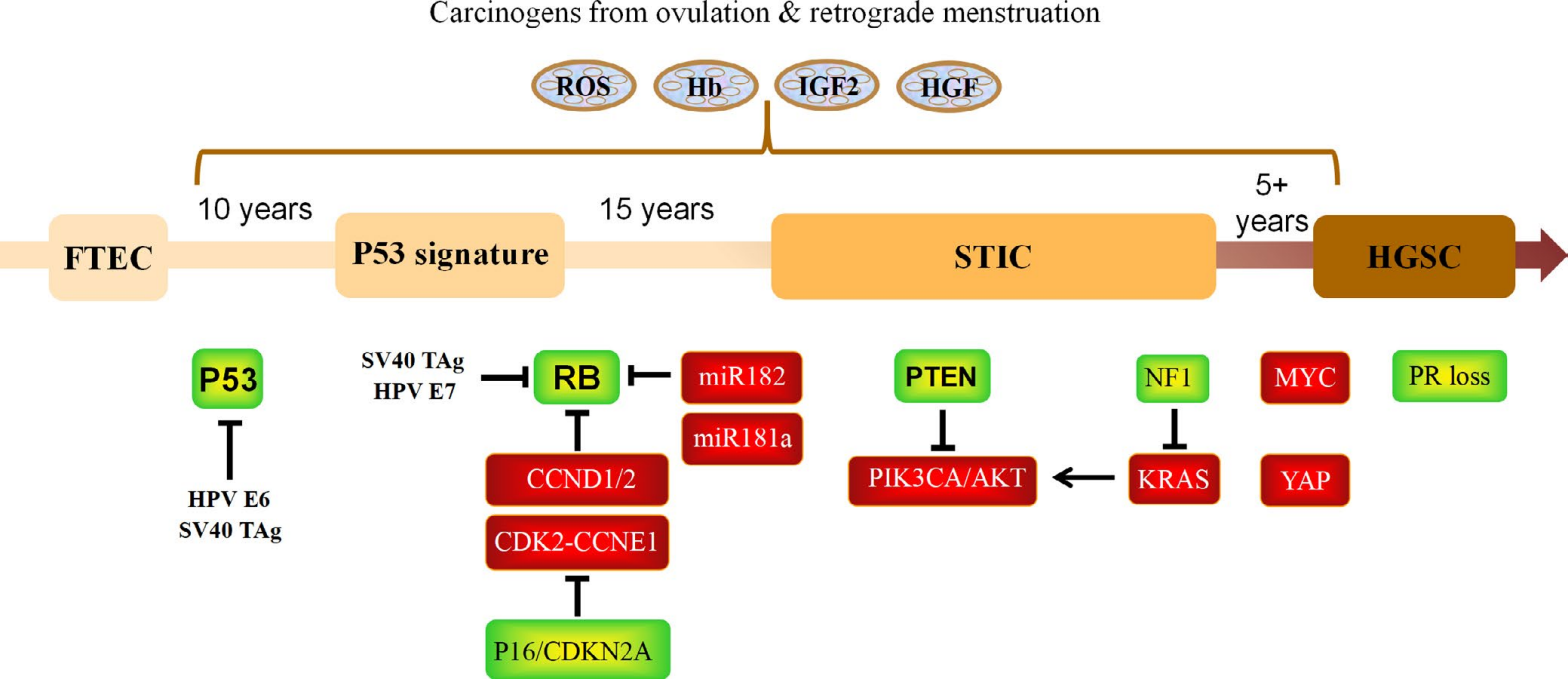
The genetic alterations and known mechanism of transformation by ovulation and retrograde menstruation in the development of HGSC from the FTE. High concentrations of ROS, Hb, IGF2 and HGF in FF are known etiological factors for inducing malignant transformation of FTECs. P53 is considered to be the first step in transformation, and the loss of RB is one of the conditions required for malignant transformation. The abnormality of PTEN often endows transformation of FTECs with the ability to form tumours in vivo. YAP and miR‐181a are the only two known independent non‐viral oncogenes that have been shown to promote tumour formation independently. Moreover, miRNA, such as miR‐181a/miR‐182, has also shown an increasingly important role in promoting malignant transformation of cells. Known etiological factors are shown in grey ovoid, overexpressed/amplified oncogene is shown in red squares, and downregulated tumour suppressor is shown in green squares. FF, ovulatory follicular fluid; FTEC, Fallopian tube epithelial cells; HGSC, high‐grade serous carcinoma; PR, progesterone receptor; STIC: serous tubal intraepithelial carcinoma

The Cancer Genome Atlas (TCGA) has globally characterized the genetic alterations in HGSC tumour samples from patients. A clear landscape of driver mutation involving genes and pathways, including TP53, RB signalling (CDKN2A, CCNE1, CCND1, CCND2 and RB1), PI3K/RAS signalling (PTEN, NF1, KRAS, PIK3CA and AKT), MYC transcription factor and DNA damage response (ATM, ATR and FA core complex) and homologous recombination repair pathways (BRCA1, BRCA2, EMSY and RAD51), has been unveiled.^75^ Among them, TP53 mutation is the earliest universal hit.^76^, ^77^, ^78^ In addition to cell cycle control, mutant p53 proteins may acquire gain of function (GOF) activity. These proteins can interact with new DNA targets and protein partners, promoting genomic instability, proliferation, invasion, metastasis, inflammation, angiogenesis and chemoresistance.^79^ Clinical data have shown that the prognosis of HGSC patients with GOF p53 mutations was poorer than that of patients with loss of function p53.^80^ Mutation or amplification of the RB pathway genes occurs in 2/3 of cases and early after TP53 change. This early dual disruption of p53 and Rb pathways underscores the DNA copy number variation and chromosomal instability phenotypes present in tubal precursor lesions early in HGSC development.^74^ Additionally, BRCA1/2 and PTEN mutations also have been found in STIC lesions,^19^ and the oncogenic roles of Yap^81^ and loss of Pten^82^ and NF1^83^ have been confirmed in genetic‐engineered mouse model. Figure 1 summarizes these genetic alterations and known mechanism of transformation by ovulation and retrograde menstruation in the development of HGSC from the FTE.

## 2 CELL MODELS USED FOR THE STUDY OF THE GENETIC ALTERATION AND MECHANISM OF HGSC DEVELOPMENT

Given the shifting paradigm of cell origin, cell lines derived from the FTE with different severities of oncogenic alterations were established to explore the molecular mechanism of cell transformation, biomarkers for early detection and prevention methods. Herein, we summarize the immortalized and transformed cell lines derived from the oviduct of humans and mice and their applications in this rapidly evolving field of research.

### 2.1 Human HGSC cell lines

Cancer cell lines used as a tumour model in vitro have had a profound impact on cancer research and greatly promoted the development of new biomarkers and targeted cancer therapies.^84^, ^85^, ^86^ However, before the molecular classification and binary stratification system was established, EOC cells could not be discerned in studies. In addition, misidentification and cross‐contamination of some cell lines have also hindered research progress.^87^ The consequence has been a prolonged delay in discovery of targeted therapies and specific biomarkers in ovarian cancer. Another problem is that some ovarian cancer cell lines do not match the characteristics of homologous tumours.^88^, ^89^

Given the knowledge of the molecular characteristics of different EOC, particularly the unique features of HGSC, that is the universal TP53 mutation and profound DNA copy variation, researchers have revisited the commonly used EOC cell lines for precise classifications. Domcke et al^90^ evaluated 47 existing ovarian cancer cell lines and compared the differences in DNA copy number changes, mutations and mRNA expression profiles with HGSC tumour samples. The researchers found that the most commonly used cell lines, such as SKOV3 and A2780, were actually not HGSCs. Instead, KURAMOCHI and OVSAHO cell lines, which were not classified as HGSC previously, had the highest correlation with molecular features of HGSC. Additionally, Anglesio et al^91^ identified some cell lines, such as CAOV3, OVCAR3, OVCAR4, OVCAR5 and OVCAR8 as HGSC cell lines. Table 1 summarized the morphology, original annotation and key cancer‐driving genes of 29 cell lines confirmed with the molecular and histological features of HGSC.

**TABLE 1:** HGSC cell lines (n = 29) with confirmed molecular and histological features of HGSC

Cell name	Morphology	Original annotation	TP53	RB1	CCNE1	TPX2	KRAS	MYC	ERBB2	BRCA1	BRCA2
59M	S	Mixed	Mu	WT	WT	Un	WT	Am	WT	WT	WT
CAOV3	E	AC	Mu	WT	WT	Un	WT	WT	WT	WT	WT
CAOV4	E	AC	Mu	WT	WT	WT	WT	Am	WT	WT	WT
COV318	E^a^/S^b^	AS^a^/SC^c^	Mu	WT	Am	WT	WT	WT	WT	WT	WT
COV362	S	EC	Mu	De	WT	Un	WT	Am	WT	Mu	WT
KURAMOCHI	E	AS	Mu	WT	WT	Un	Am	Am	WT	WT	Mu
OAW28	E	AS	Mu	WT	WT	Am	WT^a^/Am^b^	WT	WT	WT	WT
OV17R	E	AS	Mu	WT	Am	WT	WT	WT	WT	WT	WT
OV90	E	AS	Mu	WT	Am	WT	WT	WT	WT	WT	WT
OVSAHO	E	SC	Mu	De	WT	Un	WT	WT	WT	WT	Mu
PEA1	S	PE	Mu	WT	WT	WT	WT	WT	WT	WT	WT
PEA2	E	AS	Mu	WT	WT	WT	WT	WT	WT	WT	WT
PEO1	E	AS	Mu	WT	WT	WT	WT	WT	WT	WT	Mu
PEO14	E	AS	Mu	WT	WT	Am	WT	WT	WT	WT	WT
PEO4	E	AS	Mu	WT	WT	WT	WT	WT	WT	WT	Mu
SKOV6	E	Un	Mu	WT	Am	WT	WT	WT	WT	WT	WT
OVCAR3	E	AS	Mu	WT	Am	WT	WT	WT	WT	WT	WT
OVCAR4	Un	AC	Mu	WT	WT	Un	WT	WT	WT	WT	WT
OVCAR5	Un	AC	Mu	WT	WT	Un	WT	WT	‐	WT	WT
OVCAR8	S	AC	Mu	WT	WT	Un	Mu	WT	Mu	WT	WT
FUOV1	Un	SC	Mu	WT	Am	Un	WT	Am	WT	WT	WT
JHOS2	Un	SC	Mu	WT	WT	Un	WT	WT	WT	Mu	WT
JHOS4	Un	SC	Mu	WT	WT	Un	WT	WT	WT	WT	WT
SNU119	E	SC	Mu	WT	WT	Un	WT	Am	WT	WT	WT
TYKNU	Un	AS	Mu	WT	WT	Un	WT	WT	WT	WT	WT
ONCODG1	Un	Un	Mu	WT	Am	Un	Am	WT	WT	WT	WT
OVKATE	Un	SC	Mu	WT	WT	Un	WT	WT	WT	WT	WT
UWB1.289 + BRCA1	E	Un	Mu	WT	WT	Un	WT	WT	‐	WT	WT
OVSAYO	Un	Un	Mu	Un	Un	Un	Un	Un	Un	Un	Un

Note

Morphology: E, epithelial; S, spindle. Original annotation: AC, adenocarcinoma; As, ascites; EC, endometrioid cancer; PE, pleural effusion; SC, serous cancer. Gene alternation: Am, amplification, De: delete; Mu, mutation; Un: unknown; WT, white type.

^a^ PMID25230021.

^b^ PMID23839242.

^c^ MID29880891.

In addition to the genetic analysis, Papp et al^92^ further provided an integrative characterization at genomic, epigenomic and expressional levels in 45 EOC cell lines. Francis Jacob et al summarized the characteristics of 39 ovarian cancer cell lines, including growth characteristics, mRNA/microRNA expression, exon sequences, drug response for clinically relevant therapeutics and the original clinical features and site of origin. The researchers then determined that only 14 cell lines, such as OVCAR3 and COV318, considered to be high‐grade serous types. The criteria for the putative high‐grade serous origin were presence of TP53 mutation and no MSI, or with TP53 mutation and amplification of CCNE1, MYC or TPX2.^93^

Moreover, Mitra et al evaluated the tumourigenesis of 11 HGSC cell lines 90 days after subcutaneous (sc) (1 × 10^6^ cells) and intraperitoneal (ip) (5 × 10^6^ cells) injections in nude mice.^94^ They found that OVCAR3, OVCAR4, OVCAR5 and OVCAR8 grew both ip and sc tumours; CAOV3 and OVSAHO grew ip tumours only; and only OVCAR8 formed ascites reliably. OVKATE and COV362 were tumourigenic only with sc injection. All tumours from the two sites had the histological features of HGSC. Among them, OVCAR3 formed the largest sc tumours and OVCAR8 formed the largest ip tumours. Interestingly, KURAMOCHI, although sharing the most molecular features with HGSC, did not grow tumours in nude mice, but it did grow tumours in the more severely immunodeficient mice, such as in SCID^95^ and NSG.^96^ Tumours from OVCAR3, OVCAR4 and OVKATE xenografts showed intense nuclear staining for p53, PAX8 and WT1, and those from OVSAHO, CAOV3 and OVCAR8 showed strong PAX8 and WT1 and weak p53.^94^

Haley et al^97^ systematically compared the migration, invasion, proliferation, clonogenicity, epithelial–mesenchymal transition phenotypes and cisplatin resistance of eight HGSC cell lines. They found that OVCAR5, OVCAR8 and KURAMOCHI cells exhibited the most robust invasion ability, whereas the SNU119 and OVSAHO cell lines had the lowest activity. Morphologically, SNU119 had the most epithelial‐like and OVCAR8 had the most mesenchymal‐like phenotypes. The CAOV3 cell was the most sensitive, whereas the COV362 cell was most resistant to cisplatin treatment.

### 2.2 Immortalized fallopian tube secretory cell lines

To explore the pathogenesis of a cancer, cell lines deriving from the cell of origin and representing the different transformative states are fundamental and have been established in cancers, such as lung adenocarcinoma,^98^ head and neck cancer^99^ and gastric cancer.^100^ Similarly, to study the development of HGSC from the FTE, it is necessary to establish cell lines that reflect the characteristics of the original cell and the pre‐cancerous lesions. However, tubal pre‐cancerous lesions of HGSC are always minuscule and impossible to be cultured. The median size of STIC was found to be only 1.9 mm^101^ and that of p53 signature, by its definition of >12 consecutive cells with nuclear p53 staining, was as small as <1 mm. Therefore, the alternative is to immortalize the primary cells from the tissue of origin and transform them in vitro. The fimbria part of the fallopian tube as the tissue of origin of HGSC has multiple folds of significance; it is the site that is mostly affected by ovulatory carcinogens, and thus, the site that most heavily bears the inflammatory injury and requires regenerates after ovulation.^64^ Thus, it is also the site where stem cells most abundantly present and pre‐cancerous lesions are most frequently found.^102^

Following the putative sequence of driver mutations in HGSC, that is TP53 mutation, CCNE1/RB aberration,^103^, ^104^ primary FTECs were immortalized and transformed stepwise by further genetic manipulation. Moreover, the human telomerase reverse transcriptase gene (hTERT) is routinely introduced to overcome the senescence crisis. Ronny Drapkin et al were the first to transduce hTERT to FTECs and established the FT33 cell line, which underwent senescence after ~10 passages.^105^ Immortalization was achieved by turning down the TP53 and the CDK/CYCLIN/RB pathways. For instance, the large T antigen (TAg) and small T antigens of SV40 virus were transduced to FT33 cells to establish the FT33‐TAg cell line, and hTERT plus TAg was used to establish the FT194 and FT190 cell lines.^105^ Unlike SV40 that infects both monkeys and humans and does not cause human tumours, human papillomavirus (HPV) infects humans as the only host and has higher tumourigenic activity. Thus, Chu TY et al used E6/E7 oncogenes of HPV to turn down TP53/RB and introduced hTERT to establish the FE25 cell line.^51^ By sharing the same conserved motifs for the inactivation of TP53 and RB, both viruses used the same strategy to harness the host cell cycle for an unlimited proliferation, and this is exactly the same genetic changes for HGSC to initiated from the FTECs. Oncogenes of SV40 and HPV viruses have a diverse effect on host cells other than TP53 and RB downregulation.^106^, ^107^

For a more specific targeted mutagenesis, Karst et al introduced TP53 shRNA and a mutant cyclin‐dependent kinase CDK4^R24C^ to establish FT237, FT240, FT246 and FT33‐shp53‐R24C cell lines.^105^, ^108^ The R24C mutation makes the CDK4 protein insensitive to CDKN2A/P16 inhibition, leading to activation of CCND1/2 and loss of pRb function.^109^

To more specifically mimic GOF TP53 mutations in HGSOC,^110^ the FT282 cell line was established by introducing hTERT and mutant TP53^R175H^ to FTECs.^111^ In a later study, TP53 mutations at codons R273, R248 and R175 were each introduced into FT240 cells, which harbour hTERT and CDK^R24C^, to establish FT240‐R175, FT240‐R248 and FT240‐R273 cells. These GOF‐mutant p53s had additional mutational activities. Among them, p53^R175H^ promotes cell aggregation upon the detachment of FTECs by upregulating the expression of fibronectin, integrin α5 and TWIST1.^112^ The mouse homolog of p53^R175H^ promotes transformation, invasion and metastasis of EOC in mice,^113^ p53^R248W^ stimulates the invasion of ovarian cancer cells by binding to Rad21,^114^ and p53^R273H^ promotes HGSC through inhibiting lysophosphatidic acid phosphatase type six and increasing lipid secretion in FTECs.^115^

Table 2 lists these immortalized FTECs, methods of immortalization and their genetic alterations. These immortalized FTECs showed phenotypes of fallopian tube secretory cells expressing markers, such as PAX8 and WT1,^116^ and do not express ciliated cell markers, such as FOXJ1.^117^ The majority of these cells do not grow colonies in soft agar, and some showed limited anchorage independent growth (AIG), suggesting evolution of early transformed clones.^96^

**TABLE 2:** Immortalized human fallopian tube fimbrial epithelial cell lines

Cell line name	Method of immortalization	Gene alterations	PMID
FT33‐TAg	FTE+hTERT+SV40 large T plus small T	hTERT gain, P53 and RB loss	21502498
FT190	FTE+hTERT+SV40 large T	hTERT gain, P53 and RB loss	22936217
FT194	FTE+hTERT+SV40 large T	hTERT gain, P53 and RB loss	22936217
FE25	FTE+HPV16 E6/E7+hTERT	P53 and RB loss, hTERT gain	26363031
FT282	FTE+hTERT+TP53^R175H^	hTERT gain, TP53 GOF mutation	24366882
FT282‐c11	FTE+hTERT+TP53^R175H^ (Clonal)	hTERT gain, TP53 GOF mutation	30459449
FT33‐shp53‐R24C	FTE+hTERT+p53 shRNA+CDK4^R24C^	TP53 knockdown, Rb pathway loss	21502498
FT237	FTE+hTERT+p53 shRNA+CDK4^R24C^	hTERT gain, TP53 knockdown, RB pathway loss	22936217
FT240	FTE+hTERT+p53 shRNA+CDK4^R24C^	hTERT gain, TP53 knockdown, RB pathway loss	22936217
FT246	FTE+hTERT+p53 shRNA+CDK4^R24C^	hTERT gain, TP53 knockdown, RB pathway loss	22936217
FT240‐R175	FTE+hTERT+TP53^R175H^+CDK4^R24C^	hTERT gain, TP53 GOF mutation, RB pathway loss	32471985
FT240‐R248	FTE+hTERT+TP53^R248^+CDK4^R24C^	hTERT gain, TP53 GOF mutation, RB pathway loss	32471985
FT240‐R273	FTE+hTERT+TP53^R273^+CDK4^R24C^	hTERT gain, TP53 GOF mutation, RB pathway loss	32471985

Abbreviation: GOF, gain of function.

### 2.3 In vivo and in vitro transformed cell models

With immortalized FTECs available, driver mutations have been introduced and examined for their transformation capability. For example, inactivation of Rb but not Brca1, together with Trp53 inactivation, was found to be sufficient for mouse OSE transformation.^118^, ^119^ To explore the role of CCNE1, which is a negative regulator of RB and frequently amplified early in HGSC development, CCNE1 was overexpressed in TP53^R175H^‐mutated FT282 cells to derive the FT282‐CCNE1 and FT282‐V (vector control) cell pair.^112^ Compared with FT282‐V, CCNE1 overexpression greatly promoted cell proliferation,^112^ had a higher proportion of centrosome amplification^120^ and increased transformed phenotypes, including induction of AIG and xenograft tumourigenesis by ovulatory FF.^52^

A combination of Myc activation with Trp53 disruption had induced oncogenic transformation of OSE^121^ as well as in oviductal epithelial cell lines. Transduction of MYC^T58A^ or H‐RAS^V12^ into immortalized human FT33‐TAg cells both resulted in transformation with AIG and tumourigenesis phenotypes.^105^ The TP53/RB1‐disrupted cell line, FT33‐shp53‐R24C, was further transformed by knockdown of the B56γ subunit of protein phosphatase 2A (PP2A‐B56γ)^122^ and c‐MYC, which led to the fully transformed FT33‐shp53‐R24C‐shPP2A‐Myc cell line.^105^ By testing the combinations of more tumour genes, Robert et al concluded that mutations in TP53, RB1, PTEN and CDKN2A synergistically contributed to cellular transformation.^123^ TP53 is universally disrupted in HGSOC. Rb1 and PTEN mutations frequently coexist in multiple cancers, such as HGOSC and metastatic prostate cancer. *CDKN2A* encoded p14ARF or p16INK4a by alternate reading frame. These two cell cycle regulating proteins activate TP53 and RB1, respectively.^124^, ^125^, ^126^ Compared with TP53 mutations alone, Trp53/Rb1, Trp53/Cdkn2a and Trp53/PTEN combinations all improved the transformation efficiency (colony number) and colony size in OSE stem cells. The four genes, Trp53, Rb1, Cdkn2a and PTEN, had the greatest conversion efficiency when targeted simultaneously. The synergistic deficiency of Trp53, Rb1 and PTEN is considered to be a core state for the efficient translation of OSE‐SC in vitro.^123^

Robert et al tested 20 oncogenes postulated by TCGA and found most of them (LRP1B, FANCM, CREBBP, RAD51C, FAT3, APC, FANCD2 and GABRA6) did not improve transformation rates, whereas several (FANCD2, APC, FAT3 and RAD51C) actually reduced transformation. Only two genes, Ankrd11 and Wwox, enhanced the transformation frequency of Trp53‐/Cdkn2a‐/PTEN‐OSE‐SC.^123^ Deletions of both Ankrd11 and Wwox were found in genomic analysis of tumours from Trp53/Brca/PTEN‐deletion mouse models, suggesting that they may be involved in tumour initiation or progression.^22^ Both Ankrd11 and Wwox are also involved in Trp53‐related pathways. ANKRD11 binds to TP53, promotes its transactivating activity and partially restores its ability to bind to the DNA of the CDKN1A promoter.^127^, ^128^ Wwox greatly affects the response of TP53 to genotoxic stress, and downregulation of Wwox abolishes p53‐dependent apoptosis.^129^, ^130^

Considering that actin cytoskeletal disorganization is vital in metastasis of STIC cells to the peritoneum metastasis,^131^ calponin‐1 (CNN1) caught the eye of Wang et al.^132^ They found that CNN1 was downregulated in HGSC from the ovaries and the fallopian tubes compared with normal ovaries, normal fallopian tubes and fallopian tube epithelial scrapings. Immunohistochemistry also showed high expression of CNN1 in FTE, but not in ovarian HGSC tissues. Knockdown of CNN1 induced transformation of the immortalized FE25 cell line, acquiring AIG and xenograft tumourigenesis in NSG mice. In an FE25‐RAS cell line, overexpression of CNN1 significantly reduced cell motility, invasion, AIG and xenograft tumourigenesis. The results indicated that downregulation of CNN1 was necessary for anoikis survival and cell transformation, the essential step for HGSC metastasis.^132^

The YAP transcription activating protein, which mediates growth‐suppression signals downstream of various biological and environmental cues, was tested in FT194 cells. Xenograft of FT194‐YAP cells resulted in slow‐growing small subcutaneous tumours. As a comparison, FT194 carrying S127A mutant YAP resulted in rapid growing and larger tumours, which was consistent with the phenotype of HGSC.^81^, ^133^ At present, YAP signalling activation is the only known independent oncogene in non‐viral oncoproteins that have been shown to promote tumour formation by immortalizing FTSECs.

Some researchers have focused on the role of some miRNA in oncogenic transformation. Matthew Knarr et al found that miR‐181a could initiate intermittent large‐scale genomic instability and efficient tumourigenesis in FTSECs by simultaneously targeting RB1 and STING genes. Moreover, miR‐181a is believed to have the potential as a biomarker for early detection of HGSOC.^134^ Indeed, FT194 cells transfected with miR‐182 induced tumour growth in 45% (9/20) of the mice, whereas only 10% (1/10) of the mice were detected in the control group. Similar data were repeated in the FT237 cell line.^135^

Table 3 and Figure 1 present the above‐mentioned tumour‐promoting and tumour‐suppressing genes/molecules that are important for the development of HGSC and have been tested in the immortalized FTECs in vitro and in vivo.

**TABLE 3:** Malignant transformation of immortalized human FTECs

Cell line name	Genetic alterations	Methods of transformation	Manipulation	Xenograft	Tumour rate	PMID
FE25	P53/RB loss	Add driver mutations	H‐RAS overExp	NSG sc/ip	7/7, 6/6	28977852
			shCNN1	NSG sc/ip	2/3, 2/2	
			H‐RAS, CNN1 overExp	NSG sc/ip	1/8, 1/6	
		Promote clonal evolution	Control	NSG sc/ip	0/11, 0/6	30852161
			Local injection with FF	NSG sc/ip	7/11, 4/9	
			Local injection with IGF2‐depleted FF	NSG sc	2/6	
			Local injection with PAPP‐A‐depleted FF	NSG sc	2/6	
		Both	Local injection with FF, plus shIGF1R	NSG sc	0/9	
FT33‐TAg	P53/RB loss	Add driver mutations	H‐RAS overExp	Nude ip	5/5	21502498
				NSG ip	2/2	
			c‐MYC overExp	NSG ip	2/5	
FT33‐shp53‐R24C	P53 loss, CDK4^R24C^		PP2A‐B56γ loss, c‐MYC overExp	NSG ip	2/4	
FT194	P53/RB loss	Add driver mutations	Control	Nude, ib	1/10	26472020
			miR‐182 overExp	Nude, ib	9/20	
			Wild type YAP overExp	NSG sc	6/8	26364602
			YAP^S127A^ overExp	NSG sc	8/8 (larger)	
FT237	P53 loss, CDK4R24C	Add driver mutations	pscram‐miR	sc/ip	0/10, 0/8	32591511
			pmiR‐181a	sc/ip	9/10, 3/8	
			shRB	sc	17/18	
			pmiR‐181a+anti‐miR	sc	1/10	
FT282‐CCNE1	TP53^R175H^, CCNE1 overExp	Long passage	Control	NSG ip	2/8	30852161
		Promote clonal evolution	Local injection with FF	NSG ip	6/8	
		Add driver mutations	Control	ND	(AIG colony = 4)	27663592
			AKT2 overExp	ND	(AIG colony = 7)	
			AKT3 overExp	ND	(AIG colony = 8)	

Abbreviations: FF, follicular fluid; PAPP‐A, pregnancy‐associated plasmatic protein A.

### 2.4 Immortalized murine oviduct epithelial cells and OSE cells

Unlike human cells that require transgenes, such as hTERT, to overcome senescence, murine cells are readily immortalized in culture and, upon long‐term passage, may transform spontaneously. As the natural origin of transformation, OSE cells have been immortalized and transformed.^136^, ^137^ After long‐term culture of the OSE cells from FVB/N female mice,^138^ McCloskey et al established the STOSE cell line as the first spontaneous murine OSE model of HGSC.^139^ By introducing SV40 T antigen DNA and by homozygous deletion of Trp53 gene in OSE from C3H/He mouse, Kido et al^140^ constructed TAg‐MOSE and p53‐def‐MOSE cell lines, respectively. They showed that Trp53 deletion did not show any transformation phenotype, whereas TAg‐MOSE formed tumours in nude mice. The widely used ID8 cell line of C57B6 background was derived from OSE and spontaneously transformed by prolonged in vitro culture.^141^ However, the ID8 cell line does not carry driver mutations of either HGSC (Trp53, Rb, Brca1/2 etc) or endometrioid and clear‐cell carcinoma (Arid1a, Ras et al). Walton et al^142^ introduced Trp53 and Brca2 knockouts to the ID8 cells. As expected, loss of p53 markedly increased in vivo the tumour growth rate within the peritoneal cavity, whereas Brca2 knockouts introduced defective homologous recombination repair and rendered cells sensitive to PARP inhibitor‐mediated cytotoxicity.

After the discovery of oviduct as the main origin of HGSCs, murine oviductal epithelial (MOE) cell lines were established. In one successful example, MOE was pooled from multiple oviducts from 8‐week‐old female CD1 mice.^136^ The cells were then continuously cultured for up to 130 passages to generate the MOE^low^ (passages 5‐25) and MOE^high^ (passages 85‐120) cells. The MOE^low^ cells failed to develop tumours in either the sc or ip sites in syngeneic transplantation. In contrast, subcutaneous transplantation of MOE^high^ cells developed tumours within an average of 117 ± 9 days. However, the tumours showed poorly differentiated sarcoma‐like features and failed to grow any tumours upon intraperitoneal injection.^137^ The same team discovered over 100‐fold overexpression of Prl2c2 in MOE^high^ cells and suggested Prl2c2 as a driver of tumourigenesis in this system.^143^ By genetic manipulation of the driver mutations, the MOE‐based model of ovarian cancers of different histological types was established. Eddie et al^144^ tested different combinations of driver mutations in MOE cells, including shPten, Trp53^R273H^, AKT^MYR^ and KRAS^G12V^. They found silencing of PTEN resulted in HGSC with wide intraperitoneal and ovarian spreading; addition of Trp53 mutation to PTEN silencing did not enhance the transformation, whereas addition of KRAS mutation promoted in vitro transformation and reduced survival in vivo. To create oviduct cells that phenocopy the most common patient‐relevant mutations, Iyer et al^145^ introduced multiple genetic alterations to MOE cells according to human HGSC with deficient (HR‐D) or proficient (HR‐P) homologous recombination repair function. The HR‐D MOE was produced, under a Trp53^−/−D^ background, through a combined loss of Trp53, Brca1, Pten and Nf1, plus overexpression of Myc and Trp53^R172H^. The HR‐P MOE was produced (also in Trp53^−/−^ background) through overexpression of Ccne1, Akt2, Trp53^R172H^, plus KRAS^G12V^, Brd4 or Smarca4 overexpression. Table 4 summarizes the tumourigenesis outcomes in these murine ovarian cancer models based on immortalization and transformation of cells derived from the oviduct epithelium and OSE.

**TABLE 4:** Immortalized and transformed murine oviduct and ovarian surface epithelial cell lines

Cell name	Mouse strain	Methods of transformation	Altered driver genes	Tumourigenesis			PMID
				Trans‐plant target	Tumour rate	Tumour type	
Oviduct epithelial cell lines							
MOE^low^ (5‐25 passages)	CD1	Not transformed	None	Nude sc	0/5	Nil	26236688
MOE^high^ (85‐120 passages)	CD1	Long passage	Unknown	Nude sc, ip	5/5, 0/5	Sarcoma‐like tumour with some PAX8 positivity	
MOE shPTEN	FVB/N	Driver mutation	PTEN	FVB/N sc	4/4	HGSC‐like with intraperitoneal spreading	25971410
MOE shPTEN, KRAS^G12V^	FVB/N	Driver mutation	KRAS	FVB/N sc	6/6	HGSC‐like with higher tumour burden and lower survival	
PPNM HR‐intact HGSC mimic	C57B6	Driver mutation	Trp53^R172H^Pten^−/−^Nf1^−/−^Myc^OE^	C57Bl/6 ip	Nil	hosts recapitulated typical HGSC histopathology	33158843
BPPNM HR‐defective HGSC mimic	C57B6	Driver mutation	BRCA1^−/−^Trp53^R172H^Pten^−/−^Nf1^−/−^Myc^OE^	C57Bl/6 ip	Nil		
Ovarian surface epithelial cell lines							
STOSE	FVB/N	Long passage	Upregulated Ccnd1, loss Cdkn2a	FVB/N ip	4/4	HGSC‐likr	24672774
TAg‐MOSE	C3H/HE	SV40 TAg	p53/Rb	Nude sc, ip	3/8, 11/12	Undifferentiated malignancy with heterogeneous tissues	9820870
p53‐def‐MOSE	C3H/HE	Not transformed	p53 deficient	Nude sc, ip	0/6, 0/6	Nil	
ID8	C57BL/6	Long passage	Low mutation burden, no driver mutation	C57Bl/6 ip	12/12	No molecular features of HGSC, or endometrioid/Clear‐cell Ca	27530326
ID8 Trp53^−/−^	C57BL/6	Long passage	Trp53	C57Bl/6 ip	22/22 (faster growth)	More HGSC‐like	
ID8 Trp53^−/−^; Brca2^−/−^	C57BL/6	Long passage	Trp53, Brca2	C57Bl/6 ip	18/18 (better survival)	More HGSC‐like	

Note

Nil: unknown

### 2.5 IGF2 in FF promoted transformation of immortalized FTECs by inducing expansion of aneuploidic cell clones

While long‐term passage of murine cells typically results in spontaneous transformation, it rarely occurs in immortalized human cells. However, given the genomic instability caused by the p53 and Rb pathway disruptions, immortalized FTECs do have a chance to evolve transformed cell clones after long passage. For example, the HPV E6/E7 and hTERT‐immortalized FTE cells (FE25 cells) showed a subdiploid DNA and chromosome count at passage 31 with chromosome numbers ranging from 42 to 43. At passage 115, a polyploidic subpopulation with 74‐77 chromosomes arose in addition to the subdiploid population with 39‐40 chromosomes. A more extensive chromosomal polyploidy and aneuploidy were noted in both populations, especially in the polyploid one, suggesting a tendency towards transformation. Moreover, this evolution of chromosomal instability can be accelerated by the stemness activation and clonal expansion activities of IGF2; thus, the transformed clone can be selected and enriched.^47^ This clonal selection activity of IGF2 as well as its binding protein and the PAPP‐A protease for its activation (the IGF‐axis proteins) were found abundantly present in human ovulatory FF, which was collected from women undertaking oocyte retrieval for in vitro fertilization. By adding 10% FF or pure IGF2 to FE25 cells, AIG was observed, and poorly differentiated Ca arose when FE25 cells and FF were co‐injected into NSG mice intraperitoneally or subcutaneously.^52^ This transformation activity of FF was confirmed in another immortalized FTEC line FT282‐CCNE1. In agreement with the crucial role of the IGF‐axis pathway, the transformation was inhibited by shRNA or inhibitor of IGF‐1R or when IGF2 or PAPP‐A was depleted from FF.

### 2.6 Limitations and improvements of current cell models

On the one hand, the molecular and histological characteristics of the currently constructed cancer cell lines were inconsistent with the original tissues.^146^, ^147^ On the other hand, some people doubted if the cancer cell lines established in conventional media could reflect the diversity of human cancers accurately and if they could have a role in drug development.^148^, ^149^ Artificially engineered cells are difficult to construct. Under the existing cell‐culture system, primary cultured cell lines needed a long time^150^ and were difficult to grow in vitro owing to the difference between the in vitro and in vivo growth environments and culture conditions.^151^, ^152^ Current 2D cell‐culture models lack the original tissue architecture and microenvironment, with consequent loss of the expression of important hormone receptors. Moreover, it is technically difficult to derive cell lines from the minute pre‐cancerous lesions. Therefore, we need to construct more realistic cell lines to meet severe scientific challenges. Firstly, regarding the origin of cancer, different stages of cell lines need to be constructed according to the pathological origin and aetiology of the disease to deal with different stages of research rather than a single cell line throughout all research. Secondly, cell‐culture systems with appropriate conditions also need to be constructed to maintain the most original characteristics of the cell lines and enable them to characterize the original tissue.^153^, ^154^ Considering the tissue microenvironment and cell‐environment interactions, 3D organoid culture, co‐culture and other new cultural technologies that more closely simulate the in vivo environment will provide possibilities for more realistic research.^27^, ^155^, ^156^ Finally, genetic‐engineered mouse models would provide the closest genocopy and phenocopy to mimic tumorigenesis, tumour‐microenvironment and immune response to HGSC.^20^, ^21^, ^22^, ^162^

## 3 CONCLUSION

With more in‐depth research on the origin of ovarian HGSC, ovaries and FTE cells are increasingly considered to be the main origins of this cancer, and more cell lines and animal models have been constructed in the process. In this review, we summarized the currently available cell systems derived from these two origins and methods of transformation leading to the development of HGSC in both human and murine systems. These cell lines and transformation models provide a valuable basis for understanding the mechanism of malignant transformation and for research on disease prevention, early diagnosis and drug screening. Certainly, cell lines cultured in vitro or transplanted in vivo cannot fully simulate the process of disease occurrence. Different GEMs of HGSCs derived from the two tissues of origin are available. However, significant limitations still exist in the mouse in vivo model, such as the lack of menstruation and confinement of ovulatory FF within the ovarian bursa and need to be overcome.

## CONFLICT OF INTEREST

The authors declare that there is no potential competing interest.

## AUTHOR CONTRIBUTIONS

TC and JM conceived the structure of manuscript and revised the manuscript. JM and HT made the figures and table. HH, CH and NW completed a large number of preliminary studies. YL and WZ revised the manuscript. All authors approved the final manuscript.

## Data Availability

Data sharing is not applicable to this article as no new data were created or analysed in this study.
